# Detection of Pesticides in Active and Depopulated Beehives in Uruguay

**DOI:** 10.3390/ijerph8103844

**Published:** 2011-09-28

**Authors:** Lucía Pareja, Marcos Colazzo, Andrés Pérez-Parada, Silvina Niell, Leonidas Carrasco-Letelier, Natalia Besil, María Verónica Cesio, Horacio Heinzen

**Affiliations:** 1 Cátedra de Farmacognosia y Productos Naturales, Departamento de Química Orgánica, Facultad de Química, UDELAR, General Flores 2124, Montevideo 11800, Uruguay; 2 Polo Agroalimentario y Agroindustrial, Centro Universitario Paysandú, UDELAR, Ruta 3 km 363, Paysandú 60000, Uruguay; 3 INIA-La Estanzuela, Ruta 50 km 11, Colonia 70000, Uruguay

**Keywords:** honeybees, bee products, beehive depopulation, insecticides

## Abstract

The influence of insecticides commonly used for agricultural purposes on beehive depopulation in Uruguay was investigated. Honeycombs, bees, honey and propolis from depopulated hives were analyzed for pesticide residues, whereas from active beehives only honey and propolis were evaluated. A total of 37 samples were analyzed, representing 14,800 beehives. In depopulated beehives only imidacloprid and fipronil were detected and in active beehives endosulfan, coumaphos, cypermethrin, ethion and chlorpyrifos were found. Coumaphos was present in the highest concentrations, around 1,000 μg/kg, in all the propolis samples from active beehives. Regarding depopulated beehives, the mean levels of imidacloprid found in honeycomb (377 μg/kg, Standard Deviation: 118) and propolis (60 μg/kg, Standard Deviation: 57) are higher than those described to produce bee disorientation and fipronil levels detected in bees (150 and 170 μg/kg) are toxic *per se*. The other insecticides found can affect the global fitness of the bees causing weakness and a decrease in their overall productivity. These preliminary results suggest that bees exposed to pesticides or its residues can lead them in different ways to the beehive.

## 1. Introduction

During the last fifteen years, a continued depopulation of beehives has been reported in USA, Italy, France and Spain and other countries. This phenomenon which is characterized by bees suddenly abandoning beehives, with evidence of bee death, is called Colony Collapse Disorder (CCD) in the United States. Colony abandonment is a rather common event in social insects like termites. When the environmental conditions in and outside the colony are dangerous to its survival, the insects migrate. Triggers for this situation, among others, are the presence of pesticides or a lack of food that hampers colony development [1–6]. Although the scientific literature has several mentions of honey bee disappearances—in the 1880s, the 1920s and the 1960s—with descriptions that sound similar to CCD, there is no way to know for sure if the problems were caused by the same agents as today’s CCD.

Perhaps the most striking difference is the magnitude of the event and its simultaneous occurrence all over the World [5]. It transpires that there is not a single cause for the disorder, but several authors have pointed out that bad sanitary environment within and around the honeycomb is one of the most important reasons for bee loss. The beehive health can be affected by many factors including hygienic management, innate immunity, viral illnesses, pesticide sensitivity, nutrition, adult age, and temperature [7,8]. These stressors, in general, compromise the immune system of bees like other social insects and may disrupt their social organization, making colonies more susceptible to disease.

In CCD cases where environmental pollution is an important cause for bee disappearance, it has been proposed that foraging activity is the starting point for the intoxication of the colony. Forager bees harvest pollen, nectar and resins from plants growing within an average radius of 5 km from the beehive. The foraging process is a complex behavioral phenomenon of coordinated individual performances based on activities like communication dances, food exchange, and the habit of flying out to the last source to forage again [9]. The literature reports the noxious effect of sublethal doses of different pesticides on return flights, orientation, and foraging efficacy [9,10].

The colony collapse disorder (CCD) has been reported in areas where intensive agricultural practices constitute the main economic activity. Particularly in Uruguay, continuous beehives losses were recorded since 2002. After this first event, a monitoring program has been established showing that the major losses occurred in regions where soy and sunflower crops are the most important agricultural activities during summer [11].

Many insecticides are routinely used to protect these crops against herbivorous insects. The most applied ones have been imidacloprid, organophosphorus pesticides [1], endosulfan and fipronil, which are actually banned for outdoor applications. Imidacloprid is a systemic neonicotinoid insecticide, used as a sunflower seed coater [12]. The widespread use of imidacloprid as a systemic insecticide, and its possible translocation to pollen and nectar, has raised concerns for the possible detrimental impact on beneficial insects [13].

Fipronil is a systemic insecticide acting on GABA regulated receptors and it is very toxic to honey bees [14]. Moreover, it is highly toxic to other non-target insects besides honeybees and the LD_50_ to honeybees is very low (44.4 μg/kg) [15,16]. Fipronil has a long lasting action; the bees carried it to the beehive and spread the toxicant into the honeycomb. Sublethal doses can affect gustatory perception, olfactory learning, and motor function in the honeybee, lowering the global fitness of the colony [17].

Organophosphorus insecticides and endosulfan are applied within the chemical pest control programs used in soybean plantations [18,19].

The present work reports the pesticide residue findings on bees and their products (honeycomb, honey and propolis) from depopulated as well as the results on honey and propolis from active beehives placed in the affected areas, aiming to establish a preliminary relationship between the presence of pesticide and the depopulation phenomena.

## 2. Experimental Section

### 2.1. General

#### 2.1.1. Reagents and Standards

All reagents were analytical grade purchased from Sigma-Aldrich. Chromatography column 60–100 mesh Florisil was purchased from Macherey-Nagel. Pesticide standards were provided by Dr. Ehrenstorfer, Germany. The investigated insecticides are listed in Table 1.

Pesticides employed in this study were selected based on their relevance for honey bees as reflected by the frequency of residue findings in bee products derived from literature, the pesticides-online database [20] and the toxicity towards honeybees, as well as based on recent data concerning the usage of pesticides in Uruguay [21].

### 2.2. Equipment

#### 2.2.1. GC Chromatography

Gas chromatographic analyses were performed using a Shimadzu GC 17A equipped with an ECD/FPD detector and a PTV injector using an internal standard method. OCs, fipronil and pyrethroid pesticides were resolved on a SE 54 capillary column (5% diphenyl, 95% dimethylsiloxane, 25 m, 0.32 mm ID, 0.25 μm film thickness; Mega Legnano, Italy). The experimental conditions were as follows: PTV, 60 °C (0.3 min), and then 500 °C/min to 270 °C (25 min). Oven temperature, 100 °C (2 min), 100–180 °C at 5.5 °C/min, 180 °C (15 min), then 180–295 °C at 10 °C/min, 295 °C (8 min). Detector temperature, 280 °C.

OP pesticides were determined with a MEGA 68 (cyano-phenyl-methylpolysiloxane) fused silica capillary column (25 m × 0.32 mm i.d. × 0.25 μm film thickness). The experimental conditions were as follow: PTV, 60 °C (0.3 min), and then 500 °C/min to 270 °C (25 min). Oven temperature, 150 °C (2 min), 150–270 at 7.5 °C/min, 270 (12 min).

GC–MS analyses used for confirmation purposes were performed using an HP 6890 GC coupled with a HP 5973 mass spectrometer supported by reference libraries, equipped with HP-5 (5% diphenyl 95% dimethylsiloxane) bonded fused-silica capillary column (25 m × 0.25 mm i.d. × 0.25 μm film thickness).

#### 2.2.2. Gel Permeation Chromatography

Was performed in a homemade apparatus, equipped with a Perkin Elmer 250 pump, detector UV Shimadzu, SPD-6AV (λ = 240 nm) and a gel permeation chromatographic column of 15 mm diameter and 230 mm long and containing 15 g Biobeads SX300 resin.

#### 2.2.3. HPLC Chromatography

Hewlett Packard-1050-HPLC equipped with DAD detector, λ = 270 nm, with a Metaphor reverse phase column (5 μm, ODS-3, 150 × 4.6 mm).

### 2.3. Sample Collection

**Figure 1:**
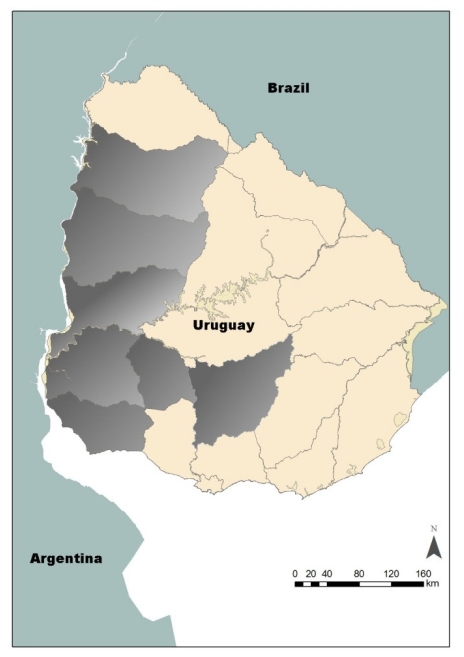
Map of Uruguay showing the sampling region (highlighted in grey).

Samples of honey, bees, honeycomb and propolis from depopulated beehives were collected by the task group from INIA La Estanzuela Agricultural Station. Samples were taken from eight apiaries affected by hive depopulation, all of them located in Western Uruguay, (Paysandú, Colonia, Rio Negro, Salto, Soriano, Flores and Florida) as shown in Figure 1, representing around 4,800 beehives in total. Samples were randomly taken, gathered together and sub-sampled at INIA La Estanzuela. These samples were subjected to imidacloprid (used in sunflower crops), fipronil and endosulfan (applied in soybean crops), and organophosphorus pesticides analyses depending on the field information of pesticide use provided by the producers.

Samples of honey and propolis from productive beehives were collected from places where no depopulation was observed in the same geographical region. The 29 apiaries sampled represented 10,000 beehives. The samples were treated as in the case of abandoned hives. The samples were subjected to organophosphorus, pyrethroid, fipronil and organochlorine insecticide residue analyses.

A diminution of bee population had been observed in the sampling region. The apiaries sampled were near soy, sunflower and *Lotus sp* plantations. Only beehives with low incidence of varroasis were considered, aiming to discard viral illnesses as possible sources of bee disappearance. Blank samples were obtained from beehives breeding near the natural woods in Maldonado department (Uruguay), were no agricultural activities were currently under way and tested for pesticide content.

### 2.4. Methods and Techniques

#### 2.4.1. Determination of Fipronil, Organochlorine and Organophosphorus Pesticides

##### Bees

Sample Preparation: Matrix Solid Phase Dispersion (MSPD) based procedure in vanishing and dead bees was followed according to previous studies [22]. One and a half grams of Florisil were blended in a mortar with 0.5 g of bee sample. The mixture was placed in a 5 mL glass syringe packed with 1.5 g Na_2_SO_4_ and 2.0 g of silica at the bottom. Fifteen mL of n-hexane were passed through and pesticides were eluted with a mixture of *n*-hexane-EtOAc (7:3). The collected eluent was evaporated until dryness with a mild N_2_ stream. Finally, 1 mL of bromophos-methyl, used as internal standard (IS) in AcOEt (1 μg/mL) was added and the solution was directly analyzed by GC-ECD/FPD and GC-MSD for confirmation purposes.

##### Honey

Sample preparation: a modified procedure based on liquid-liquid partitioning was followed, as previously reported [23]. Ten grams of raw honey were diluted in 20 mL of water. The mixture was partitioned with (3 × 10 mL) portions of EtOAc in a 50 mL separating funnel. The solution was dried under vacuum and redissolved in an n-hexane:AcOEt (1:1) mixture. Further clean-up was performed in a 5 mL glass syringe packed with 2 g Florisil eluting with 2 portions of 10 mL of an *n*-hexane-EtAcO (1:1) mixture. Solvent was evaporated until dryness with a mild N_2_ stream. One mL of bromophos-methyl (IS) in EtOAc (1 μg/mL) was added and the solution was directly analyzed by GC-ECD/FPD and/or GC-MSD.

##### Propolis and Honeycombs

For the analysis of propolis and honeycomb samples, 3.0 g of raw matrix were broken up in a mortar and 20 g of Celite were added with thorough mixing. The mixture was put into a glass column and 100 mL of dicholoromethane were passed through. The eluted solution was evaporated under reduced pressure and the residue seeded in a chromatography column containing 10 g of Biobeds X300. EtOAc was passed at a 2 mL/min flow. The eluates between 4 and 10 min were collected, the solvent evaporated and the residue redissolved in 1 mL bromophos-methyl (IS) in EtOAc (1 μg/mL) and GC-FPD analyzed and the confirmation was performed by GC-MS [24].

#### 2.4.2. Determination of Imidacloprid

Sample preparation: All the matrices (bees, honeycombs, propolis and honey) were processed according to [25]. These methodologies were adjusted to the laboratory conditions.

HPLC Analysis: Isocratic: mobile phase MeCN/H_2_0, 20:80 (AcOH 100 mg/L), 1 mL/min. Imidacloprid was identified by a combination of retention time (Rt = 12.52 min.) and UV spectrum match with respective library data.

### 2.5. Recovery Tests, LODs and LOQs

For the recovery tests, blank samples were taken from places where no beehive depopulation was observed. The material was tested for pesticide content and the pesticide free ones were employed for the recovery tests. Two different recovery tests were performed; one for honey samples and the other for the lipophilic matrices.

Blank samples were spiked with dilutions of acetonitrile solutions of imidacloprid, fipronil, organophosphorus and organochlorine compounds at 0.5 and 1.0 mg/kg concentration level. Fortified samples were prepared by mixing the pesticide solution with the blank samples. The mixture was blended to insure homogeneity, and left at room temperature until complete solvent evaporation. The spiked and the real samples were analyzed with the same analytical techniques. The reported results for each real sample were averages of two replicates, whereas for recovery studies five replicates were assayed. Table 2 shows the percentage of recoveries, the relative standard deviation and a summary of the analytical parameters of the detected pesticides in the analyzed samples at 1.0 mg/kg.

### 2.6. Data Treatment

The identification of the GC-amenable compounds was performed through retention index by selective detection whereas confirmation was carried out by injection of pure standards and comparison of their retention index and relevant selected ion monitoring of MS spectra [1]. GC-amenable compounds were quantified by calculating their relative response factor to bromophos-methyl (IS). Moreover, matrix effect was evaluated for the different matrices for the detected pesticides. Matrix effect was obtained after measuring the peak area for each pesticide at 0.5 mg/kg in solvent and in matrix respectively and calculated using the formula A_PM_-A_PS_/A_PS_.

As it was explained in section 2.5.2. imidacloprid was identified by a combination of retention time and UV spectrum match with respective library data. Due to the detection system used for imidacloprid analyses, the matrix effect for imidacloprid was not relevant; therefore the linearity was evaluated only in solvent. Calibration curves for the determination of imidacloprid were prepared in solvent at five concentration levels in the 0.1 to 1.5 mg/L range. Imidacloprid presented good linearity, with a correlation coefficient of 0.9998.

## 3. Results

### 3.1. Recoveries, LOD, LOQs and Matrix Effects

A summary of the analytical parameters of the pesticides found in the studied samples is shown in Table 2.

### 3.2. Pyrethroids, Fipronil, Organochlorine and Organophosphorus Pesticides

The pesticide residues found in the real honey samples are presented in Table 3. Chlorpyrifos and cypermethrin were the most frequently found with the highest concentrations ranging from 30 to 80 μg/kg. Fipronil and α+β endosulfan were detected in two of the twenty nine samples whereas coumaphos was found in only one honey sample. Moreover, the combination of coumaphos and chlorpyrifos was also detected in one honey sample from active beehives. Chlorpyrifos was detected along with cypermethrin in six samples and once in the same sample with ethion. Fipronil residues in honey of abandoned beehives coming from areas with soybean plantations where this insecticide was sprayed were detected at 40 and 100 μg/kg. In these samples fipronil was also detected in dead (170 μg/kg) and vanishing bees (150 μg/kg).

In propolis from active beehives, the most frequently found OP was coumaphos which was present in the 9/9 analyzed samples, followed by chlorpyrifos which was present in 7/9 of the samples and the less frequent was ethion, which had 2/9 positive findings. The levels detected ranged from 1,000–600 μg/kg for coumaphos (av. 834 ± 135 μg/kg), 150–70 μg/kg for chlorpyrifos (av. 91 ± 20 μg/kg), and 30–50 μg/kg (av. 40 ± 10 μg/kg) for ethion.

### 3.3. Imidacloprid

Imidacloprid was investigated only in depopulated beehives located near sunflower crops where this insecticide was the only pesticide applied. Honeycomb, propolis, honey and bees matrices were investigated for imidacloprid content (Table 4).

Imidacloprid was detected neither in honey from affected beehives nor in dead bees collected around them. However, it was detected in three of the honeycombs of depopulated beehives and in two samples of propolis at levels far above the LOQ. OPs, fipronil, endosulfan and pyrethroids were not detected in these samples.

## 4. Discussion

In the present work the analysis of lipophilic matrices (bees, propolis and honeycombs) and a high water content matrix (honey) were performed. Methods for the analysis of OP pesticide residues in honey are well developed, but there are few reports of clean up methodologies for propolis and honeycomb. Propolis is a resin-like material which is used by bees for sealing the beehive. It is collected in the field from different sources and deposited in the hive after a chain of cooperative work of different specialized bees. Although both honeycomb and propolis are lipophilic, their average polarity is very different. Whereas honeycomb is a non-polar matrix with high molecular weight esters (MW > 700) as main constituents, the polarity range of propolis compounds is wider and their molecular weight much lower. Flavonoids, terpenoids and phenolic acids, which are common components in propolis, have an intermediate polarity. Nevertheless, the GPC method developed here for the determination of lipophilic pesticides proved to be suitable for the clean up step in the analysis of bees, propolis and honeycombs, even though the average molecular weight of propolis constituents and OP pesticides is similar. GPC on Biobeads gave clean extracts, with little matrix effect, due perhaps to the differences in polarity as discussed above.

### 4.1. Pyrethroids and Organophosphorus Pesticides

Coumaphos is an acaricide widely used against *Varroa destructor* and its presence in the beehive is due to the specific treatment applied to them. On the other hand, chlorpyrifos is not employed in the management of the beehives, but surprisingly its residues were the most frequently detected in honey. Chlorpyrifos is the most employed OP in crop management in Uruguay and it clearly came from the environmental pollution outside the hive. Coumaphos, ethion and chlorpyrifos had also been found in Uruguayan commercial propolis samples [27]. Also in this work coumaphos, chlorpyrifos and in a lower extent ethion were detected in raw propolis. On the other hand, chlorpyrifos was found to occur in many samples, in agreement to reports by Mullin *et al*., in the USA [8]. They found chlorpyrifos in 19 of 36 honey samples of active beehives and could not evidence any direct relationship to behavioural changes.

Regarding bees, other authors also found residues of OPs, including chlorpyrifos, in samples of dead bees associated to suspected cases of bee poisoning [8,28,29] but in this work, no OP residues were found in bees.

Cypermethrin is widely used to control ants and in outdoor applications to protect crops. Its toxicity to honey bees has been known for 20 years. According to literature this insecticide is highly toxic to honey bees in laboratory tests, but field application at the recommended dosage do not put hives at risk. The LD_50_ for bees at 24 hours oral is 655.6 μg/kg [14]. In this work cypermethrin was found in 19% of the analyzed samples of honeys at concentrations levels below the LD_50_.

Cypermethrin residues were found in six of the twenty nine honey samples from active beehives. The concentration levels were below the LD_50_. Sublethal concentrations of pyrethroids have also been studied and found to produce harmful effects on honeybees such as behavioural and physiological changes [17,30,31].

It has been proved that the combination of pyrethroids and miticides acts synergically as toxic effects were detected at low concentrations suggesting that the saturation of the bees detoxification system was overloaded causing detectable noxious effects [32].

### 4.2. Organochlorine Pesticides and Fipronil

In this work, two samples of honey (Table 3) and bees belonging to the same apiary near soybean crops were contaminated at levels higher than the LD_50_ of fipronil. Fipronil was the only pesticide found in dead and vanishing bees at 170 and 150 μg/kg respectively. These results can be directly correlated to bee poisoning [15]. Acute fipronil intoxication above LD_50_ produced mortality, but also it was experimentally shown that it produces disorientation behaviour of collected bees [8,15]. Nowadays fipronil use in Uruguayan soybean crops is forbidden since the introduction of new regulation [33]. No endosulfan, OPs and pyrethroids were detected in these samples.

### 4.3. Imidacloprid

Imidacloprid residue levels found in honeycomb samples from depopulated beehives are on the same order of magnitude of what has been reported as responsible for disorientation or other behavioural disorders in bees [34–36], these results show that bees were in contact with this pesticide. This finding could suggest that imidacloprid is first ingested and then secreted with beeswax while bees build up the honeycomb.

On the other hand, its presence in propolis samples allows the assumption that bees transport imidacloprid, which is finally deposited in the beehive. For those reasons is possible to find its residues in different beehive products. Imidacloprid is used as a sunflower seed dressing agent and therefore, its most plausible origin in the cases under study is the sunflower plant. It has been reported in literature that imidacloprid contaminates all the parts of sunflowers at 1–20 μg/kg range [37]. Particularly, the flowers are contaminated at an average level of approximately 10 μg/kg at the time of foraging [38].

Moreover, average levels of few μg/kg of imidacloprid were measured in pollen and in sunflower nectar [39,40]. In 2006, Chauzat *et al*., reported residues of imidacloprid in nectar and pollen at levels that are potentially dangerous to bees [40], while Schmuck *et al*.detected no residues [2]. The presence of imidacloprid from sunflowers has been controversial, but nowadays the recent improvements in the analytical methodology allow the detection of low levels of imidacloprid, levels that were unreachable at the beginning of the century.

It has been assumed that the homing ability and behaviour of forager bees may be severely affected by imidacloprid residues [13]. Furthermore, this assumption is supported by reports of Italian and French researchers, who found that agrochemicals greatly influence bee behaviour and their feeding activities. Imidacloprid has an antifeedant/repellent effect but not a lethal or “knock-down” effect [3,12] at concentrations 10 times lower than its LD_50_. Sublethal effects of 24 μg/kg of imidacloprid in sucrose solution were noticed on olfactory learning performance [3], and other neurological effects can be detected at 6 μg/kg mainly in bees’ orientation ability while visiting a feeding source [9]. Although Nguyen *et al*., suggested that imidacloprid seed-treated maize has no negative impact on honey bees [41].

Usually, the relationship between the presence of imidacloprid and the toxic effect on bees has been restricted to the concentration in the flowering parts of the plant. Recently it has been proved that gutation drops from sunflower seedlings contains relative high amounts of imidacloprid and therefore other parts of the plant can be a source of this pesticide [42]. Our results point in the same direction as propolis it is not collected from the flowers. Resin sources for propolis are the buds of trees and exudates from plants. The resins are transported in the mouth and legs of the foraging bees and passed in the hive where bees mix the resin with wax and seal the hive. Sunflower produces a complex resin of phenolics and terpenic compounds and it is possible to assume that sunflower resin, due to the abundance of the plant in a plantation, is a forage source for propolis [43,44]. Therefore, it could be suggested that not only the flowers but also other parts of the plant, and even other plants, from where the bees collect propolis have to be considered and evaluated as a source of pesticide contamination. The presence of imidacloprid in propolis suggests both a systemic and a contact mode of action of the pesticide. Two different processes can be noticed: first, imidacloprid’s presence in the beehive means that worker bees are able to adapt to limited levels of imidacloprid in their bodies, which is excreted with the wax afterwards. Second, the presence of imidacloprid in propolis is a signal that the pesticide is included in the hive after a chain of events where not only a systemic route can be inferred but also a contact one is working, probably, leading to bee intoxication. It should be pointed out that imidacloprid was the only pesticide detected in these abandoned beehives.

Summarizing, in samples from depopulated beehives, imidacloprid and fipronil were detected. On the other hand, cypermethrin, endosulfan, chlorpyrifos and coumaphos were detected in honeys from productive beehives. From these results, the reasons for beehive depopulation in Uruguay cannot be attributed only to the presence of sublethal doses of agrochemicals in bees or their beehives, as not all the samples from the abandoned honeycombs contained pesticides. Notwithstanding, due to the overwhelming evidence of the effects of very low concentrations of imidacloprid on bees behaviour [34–37], the imidacloprid levels found in these samples could be related to the bee disappearance phenomenon from beehives where this pesticide was found, as no other insecticide was detected in these samples. It has been reported that imidacloprid turns worker bees more aggressive [12]. The presence of imidacloprid residues in honeycombs and propolis demonstrated that they are also in contact with this insecticide as they lay the agrochemical in the whole honeycomb, before abandoning it. Such behaviour could be the visible sign of the studied insecticide action on these bees [45].

In this context, the role of agrochemicals on beehive depopulation can be understood as a consequence of neurological disorders that affect forager bee orientation, caused by their repeated exposure to sublethal doses of insecticides [12,15,16].

## 5. Conclusions

Imidacloprid was detected in propolis and honeycombs, whereas fipronil was detected in honey and dead and vanishing bee samples from depopulated beehives from two different depopulation events at levels reported to cause lethal and sublethal effects to bees. OP pesticides and endosulfan were also detected at low levels in productive beehives.

Hive depopulation and the overall dropping down of its productivity are a complex phenomena and a direct correlation with pesticide residue findings cannot be simply established. The presence of some pesticides like imidacloprid and fipronil used in Uruguayan sunflower and soybean crops, respectively, could have caused bees to leave the honeycomb and acute toxicity reasons, but for the particular case of OPs, cypermethrin and endosulfan, more evidence is needed to establish a direct relationship to bee disappearance.

## Figures and Tables

**Table 1:** Target pesticides with their corresponding detection method (Positive findings were confirmed through GC/MS).

Pesticides Searched	Detection method
Aldrin	GC-ECD
Bifenthrin	GC-ECD
Bromopropylate	GC-ECD
λ-Cyhalotrin	GC-ECD
Cypermethrin	GC-ECD
Chlorfenvinphos (E+Z)	GC-FPD
Chlorpyrifos	GC-FPD
Coumaphos	GC-FPD
p,p′DDE	GC-ECD
p,p′DDT	GC-ECD
Deltamethrin	GC-ECD
Diazinon	GC-FPD
Endosulfan α+β	GC-ECD
Endosulfan SO_4_	GC-ECD
Ethion	GC-FPD
Fenvalerate	GC-ECD
Fipronil	GC-ECD
Fipronil sulphide	GC-ECD
τ-Fluvalinate	GC-ECD
Imidacloprid	LC-DAD
Lindane	GC-ECD
Malathion	GC-FPD
Metidathion	GC-FPD
Parathion metyl	GC-FPD
Tetradifon	GC-ECD

**Table 2:** Analytical parameters of the pesticides found in the studied samples.

Pesticide	Matrix	%Recovery (%RSD)	LOD (μg/kg)	LOQ (μg/kg)	ME *%
Chlorpyrifos	Honey	73.2 (12)	4	15	61
Coumaphos	Honey	79.4 (11)	12	39	78
Cypermethrin	Honey	122.1 (16)	15	50	−21
α+β Endosulfan	Honey	59.1 (21)	15	50	19
Fipronil	Honey	88.4 (9)	8	25	20
Fipronil	Bees	92.6 (22)	6	20	–
Chlorpyrifos	Propolis	123.0 (4)	9	30	−41
Coumaphos	Propolis	106.0 (12)	26	88	14
Ethion	Propolis	115.0 (6)	5	15	−49
Imidacloprid	Propolis	98.0 (13)	5	15	–
Imidacloprid	Honeycomb	98.0 (13)	5	15	–

* Matrix Effect.

**Table 3:** Frequency and levels of pesticide residues detected in honey samples [8,14–16].

Detected compounds	Chemical class	Beehive Status	Positive honey samples/analyzed samples	Highest level detected (μg/kg)	Lowest level detected (μg/kg)	Average of detected concentration (μg/kg) (SD)	LD_50_ bees (μg/kg) *
Chlorpyrifos	Ops	A	13/31	80	30	46 (12)	1333
Coumaphos	Ops	A	1/31	60		60	51444
Cypermetrhin	Pys	A	6/31	80	<LOQ	61 (14)	655.6
Fipronil	Phenyl-pyrazole	D	2/31	100	40	70	44.4
α+β Endosulfan	OCs	A	2/31	<LOQ	<LOQ	–	87444

A: Active; D: Depopulated; S.D: standard deviation;

* Assuming that a worker bee weighs 90 mg [26].

**Table 4:** Concentrations of imidacloprid in honeycombs and propolis. A report of Not Detected means that no pesticide was found with a concentration higher than the limit of detection (LOD).

Honeycomb samples	Imidacloprid (μg/kg)	Propolis samples	Imidacloprid (μg/kg)
1a	N.D.	1b	N.D.
2a	N.D.	2b	N.D.
3a	N.D.	3b	N.D.
4a	240	4b	100
5a	440	5b	20
6a	450		
